# Benign and tumor parenchyma metabolomic profiles affect compensatory renal growth in renal cell carcinoma surgical patients

**DOI:** 10.1371/journal.pone.0180350

**Published:** 2017-07-20

**Authors:** Barak Rosenzweig, Nimrod D. Rubinstein, Ed Reznik, Roman Shingarev, Krishna Juluru, Oguz Akin, James J. Hsieh, Edgar A. Jaimes, Paul Russo, Katalin Susztak, Jonathan A. Coleman, A. Ari Hakimi

**Affiliations:** 1 Urology Service, Department of Surgery, Memorial Sloan Kettering Cancer Center, New York, New York, United States of America; 2 Department of Molecular and Cellular Biology, Harvard University, Cambridge, Massachusetts, United States of America; 3 Computational and Systems Biology Program, Memorial Sloan Kettering Cancer Center, New York, New York, United States of America; 4 Renal Service, Department of Medicine, Memorial Sloan Kettering Cancer Center, New York, New York, United States of America; 5 Body Imaging Service, Department of Radiology, Memorial Sloan Kettering Cancer Center, New York, New York, United States of America; 6 Human Oncology and Pathogenesis Program, Memorial Sloan Kettering Cancer Center, New York, New York, United States of America; 7 Renal Electrolyte and Hypertension Division, Department of Medicine, University of Pennsylvania, Philadelphia, Pennsylvania, United States of America; Sun Yat-sen University, CHINA

## Abstract

**Background and objectives:**

Pre-operative kidney volume is an independent predictor of glomerular filtration rate in renal cell carcinoma patients. Compensatory renal growth (CRG) can ensue prior to nephrectomy in parallel to tumor growth and benign parenchyma loss. We aimed to test whether renal metabolite abundances significantly associate with CRG, suggesting a causative relationship.

**Design, setting, participants, and measurements:**

Tissue metabolomics data from 49 patients, with a median age of 60 years, were previously collected and the pre-operative fold-change of their contra to ipsi-lateral benign kidney volume served as a surrogate for their CRG. Contra-lateral kidney volume fold-change within a 3.3 +/- 2.1 years follow-up interval was used as a surrogate for long-term CRG. Using a multivariable statistical model, we identified metabolites whose abundances significantly associate with CRG.

**Results:**

Our analysis found 13 metabolites in the benign (e.g. L-urobilin, Variable Influence in Projection, VIP, score = 3.02, adjusted p = 0.017) and 163 metabolites in the malignant (e.g. 3-indoxyl-sulfate, VIP score = 1.3, adjusted p = 0.044) tissues that significantly associate with CRG. Benign/tumor fold change in metabolite abundances revealed three additional metabolites with that significantly positively associate with CRG (e.g. p-cresol sulfate, VIP score = 2.945, adjusted p = 0.033).

At the pathway level, we show that fatty-acid oxidation is highly enriched with metabolites whose benign tissue abundances strongly positively associate with CRG, both pre-operatively and long term, whereas in the tumor tissue significant enrichment of dipeptides and benzoate (positive association), glycolysis/gluconeogenesis, lysolipid and nucleotide sugar pentose (negative associations) sub-pathways, were observed.

**Conclusion:**

These data suggest that specific biological processes in the benign as well as in the tumor parenchyma strongly influence compensatory renal growth.

## Introduction

Chronic kidney disease (CKD) is a recognized major public health problem, associated with substantial morbidity and mortality.

Renal parenchyma volume, estimated by imaging, has been shown to correlate well with kidney function [1]. Furthermore, pre-operative kidney volume was found to be an independent predictor of glomerular filtration rate (GFR) in renal cell carcinoma (RCC) surgical patients [2]. Gain of kidney volume in the form of CRG is a well-described phenomenon that follows kidney resection and influences long-term (LT) kidney function. E.g., following donor nephrectomy, a 20% or higher increase in renal volume at 1 week was correlated with favorable renal function adaptation at 1 year [3]. Following unilateral nephrectomy, a 9.3% increase in the contralateral kidney parenchymal mass was found to be correlated with over 20% increase in GFR [4]. In the setting of renal tumor resection, over 20% and 10.9% increases in contra-lateral volumes were described following radical and partial nephrectomy, respectively [2]. Studies not only support that kidney recovery following surgical resection can occur several years out but additionally suggest that compensatory processes ensue even prior to nephrectomy and correlate with tumor size [5,6]. These suggest that CRG and tumor growth occur simultaneously in RCC patients.

Metabolomics, the systematic analysis of chemicals and small molecules, is a highly promising tool in kidney disease and cancer research [7,8]. In this study we used metabolomics to profile benign and malignant human renal tissues, in order to elucidate relevant metabolic processes affecting CRG.

**Fig 1:**
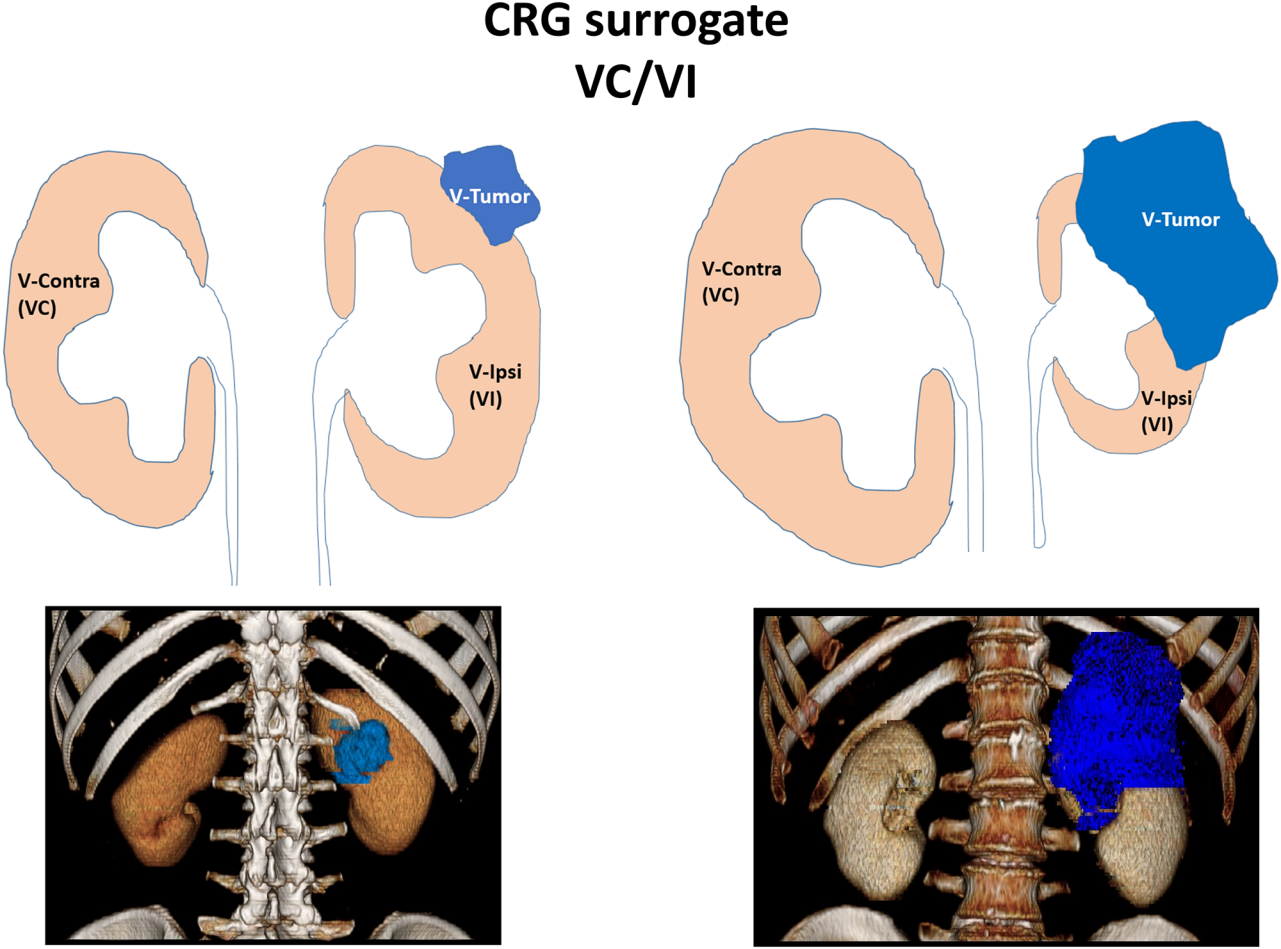
Compensatory renal growth surrogate- contra vs. ipsi-lateral benign kidney parenchyma volume. Left upper panel represents a VC/VI fold change of ~1, i.e., a surrogate to no compensatory renal growth (CRG). Right upper panel represents a VC/VI fold change higher than 1, i.e., a surrogate to higher CRG. Lower panels show representative 3D images (Aquarius iNtuition Edition software, TeraRecon Inc. CA, USA). VC- Volume of contra lateral kidney, VI- Volume of ipsi-lateral kidney (tumor side), V-Tumor- tumor volume. Lower right 3D image was horizontally flipped to correspond to the illustration.

To this end, we compiled a cohort of 49 patients with a median age at nephrectomy of 60 years with CRG as well as benign and tumor tissue metabolomics data (Table 1 and S1 Table). Contra/ipsi renal parenchyma volume was defined as pre-operative CRG surrogate, calculated using imaging and 3D software (Fig 1). Post-operative LT-CRG was calculated for radical nephrectomy patients using the remaining kidney volume change.

**Table 1:** Clinical characteristics of the cohort.

			p-value	FDR p-value
Age (mean (stdev, range))		60 (+/-11.6, 37–86)	0.44	0.46
Gender	Male	39 (79.6%)	0.43	0.46
	Female	10 (20.4%)	#	#
Race	White	43 (87.7%)	0.15	0.30
	Afro-american	5 (10.2%)	#	#
	Other	1 (2%)	0.33	0.44
PreOp GFR (mL/min per 1.73m-2)(mean, stdev)		69 (+/- 16.35)	0.36	0.44
Diabetes		9 (18.4%)	0.17	0.31
BMI (kg/m-2) (mean, stdev)		30.9 (+/- 6.55)	0.07	0.28
Hyperlipidemia		20 (40.8%)	0.47	0.47
Coronary artery disease		7 (14.3%)	0.13	0.30
Hypertension		31 (63.3%)	0.01	0.16
Smoking		25 (51%)	0.19	0.32
	pack years (mean, stdev)	8.7 (+/-12.7)		
Tumor volume (cc) (mean, stdev)		233.7 (+/- 224.1)	0.02	0.16
Primary Tumor (T stage)	pT1a	6 (12.2%)	#	#
	pT1b	5 (10.2%)	0.44	0.46
	pT2a	4 (8.2%)	0.11	0.30
	pT2b	0	NA	NA
	pT3a	10 (20.4%)	0.12	0.30
	pT3b	22 (44.9%)	0.24	0.39
	pT4	2 (4.1%)	0.04	0.20
Furhman nuclear grade	1	0	NA	NA
	2	17 (34.7%)	#	#
	2~3	1 (2%)	0.34	0.44
	3	25 (51%)	0.27	0.41
	4	6 (12.3%)	0.02	0.16
Regional lymph node (N stage)	pNx	18 (36.7%)		
	pN0	29 (59.2%)		
	pN1	2 (4.1%)		
Distant metastasis at presentation (M stage)	M0	47 (95.9%)		
	M1	2 (4.1%)		
AJCC stage	1	11 (22.4%)	#	#
	2	4 (8.2%)	0.07	0.28
	3	32 (65.3%)	0.10	0.30
	4	2 (4.1%)	0.02	0.16
Nephrectomy type^	Radical	32 (65.3%)		
	Partial	17 (16.7%)		
Imaging^	CT	46 (93.9%)		
	MRI	3 (6.1%)		

Stdev- standard deviation, PreOp GFR- pre operative glomerular filtration rate, BMI- body mass index, AJCC- American Joint Committee of Cancer. P-value represents univariate analysis calculated for CRG, FDR p-value represents significance corrected for false discovery rate. N and M stage were included in the AJCC stage association analysis. Nodes and metastasis stage were included in the AJCC stage analysis.

^Nephrectomy type, and imaging were not included as covariates.

^#^- defined as reference.

## Materials and methods

### Data collection

Data collection followed patient written informed consent and Memorial Sloan Kettering Cancer Center (MSK) institutional review board approval. For the current analysis we used data collected as previously reported in our recent publication [8]. Briefly, for kidney tissue metabolomics 138 matched/pairs of RCC tumor and adjacent benign kidney tissue from partial or radical nephrectomies performed at MSK (New York) were obtained by and stored at the MSK Translational Kidney Research Program. Samples were fresh frozen and stored at -80°C prior to metabolomic characterization. Eight hundred and seventy seven metabolites, 577 of them identified and hence for which a sub-pathway annotation exists, were quantified at Metabolon Inc. (Durham, NC) using non-targeted metabolomics gas and liquid chromatography coupled to a mass spectrometry approach.

Abundances of metabolites were median normalized and those which fell below the limit of detection were imputed as the minimal median normalized value among the cohort of patients. However, any metabolite for which more than 75% of its measurements from the cohort of patients were below the limit of detection was eliminated from all further analyses. As a result, 736 and 724 of the 877 metabolites measured in the benign and tumor tissues, respectively, were retained.

Out of a cohort of 138 patients we randomly picked 52 patients with pre- and post-operative imaging (CT scan or MRI). CT and MRI examinations were performed using the standard clinical abdomen–pelvis imaging protocols. Cross-sectional images were exported to Aquarius iNtuition Edition software (TeraRecon Inc. CA, USA). The software calculated 3D right and left renal parenchymal volume. Then, subsequent to manual rendering of the tumor area by the observer, the software automatically calculated the 3D tumor volume. Benign renal tissue was defined as the normally enhanced areas. We excluded non-parenchymal tissues, such as renal sinus fat, the collecting system, and non-enhancing cysts. Contra-lateral kidney volume (VC- Volume Contra), benign kidney volume on the tumor side (VI- Volume Ipsi), and tumor volume (V-Tumor) were calculated (Fig 1). We defined CRG as VC/VI (Fig 1). Forty two corresponding kidney and tumor volumes were independently measured by two observers who were blinded to the patient’s clinical information. Inspection of the fold change in CRG among the two observes revealed three patients with extreme volume measurement deviations (>1.25 or < 0.75, S3 Fig) and these patients were thus removed from all further analyses. For the remaining 39 patients, we took the average CRG measured by the two observes. Since three out of 42 observations with extreme deviations between the observers constitute a small fraction (7.14%) we felt confident to add ten additional corresponding kidney and tumor volumes only measured by a single observer. LT imaging data were available for 22 patients which underwent radical nephrectomy. We thus defined LT-CRG as the fold change of VCs measured at a long term follow up time point (median 3.3 +/- 2.1 years) and at the baseline time point.

Patient and disease associated metadata (covariates) including patient characteristics, comorbidities, tumor pathologic and clinical stage, and nuclear grade were also obtained (Table 1). All samples were reviewed by two expert genitourinary pathologists.

### Statistical analyses

Our aim was to find benign and tumor tissue metabolite abundances and/or patient and disease covariates which are significantly associated with CRG variation and may hence suggest a causative relationship. As this study uses retrospective data, the small sample size was pre-determined and hence the number of independent variables dramatically exceeds the number of dependent variables. As a result, a standard linear model would be inadequate to account for the collinearities in this high-dimensionality. Therefore, we sought for a multivariable regression model appropriate for this task. We chose the orthogonal partial least squares (OPLS) as it benefits from the strength of principal components analysis (PCA) regression on the one hand, and on the other hand its results are easier to interpret than those obtained by partial least squares (PLS) regression analysis [9]. Specifically, we converted the categorical covariates (Table 1) to dummy variables, combined them with either the benign or tumor metabolite abundances, centered them, and fit the OPLS model implemented in the ropls Bioconductor package [10] to them with CRG defined as the response. We used the variable influence in projection score (VIP), reported by the OPLS analysis, as a measure of the importance of the variable (metabolite or covariate) on the response. As a reference we also fitted a univariable regression linear model to CRG versus each covariate and metabolite independently. Model performance was assessed using an eight-fold cross validation analysis and in addition the OPLS model was fitted to 1,000 random permutations of the CRG order (i.e., a permutation test) to obtain a measure of random model performance. In addition, we report the R^2^ (the proportion of the variance in the dependent variable that is predictable from the independent variable) and Q^2^ (R^2^ obtained for the held out data in the cross validation analysis) of each model fit, although their informativeness is limited due to the high number of variables versus the low number of samples in our data [11]. Comparison of the OPLS VIP scores and univariable regression FDR adjusted p-values revealed a negative relationship, as reported in the metabolomics analysis by Thévenot et al. [10] (S4 Fig). We thus define independent variables with VIP score > 1 and FDR adjusted p < 0.05 as variables which are significantly associated with CRG.

To test for enrichment of biological sub-pathways among metabolites which are significantly associated with CRG we ranked metabolites by their VIP scores in descending order and performed a term enrichment analysis according to the gene set enrichment analysis algorithm [12,13], implemented in the Bioconductor piano package. This analysis reports both terms enriched with positive and negative associations with CRG at the top of the list (similar to upregulated and downregulated genes in a genes set enrichment analysis).

To test whether the negative association of tumor tissue p-cresol sulfate abundance with CRG is stronger than of its benign tissue positive association with CRG we fitted the following linear model: CRG* ~ p-cresol sulfate _tumor_* + p-cresol sulfate _benign_*, where * indicates detrended values as obtained by the R pracma package, and compared the absolute values of the p-cresol sulfate _tumor_* + p-cresol sulfate _benign_* estimated coefficients.

## Results

### Comorbidities & CRG

Hypertension was found to have a significant negative association with CRG, while tumor volume, pathologic T4 stage, Fuhrman nuclear grade 4, and American Joint Committee of Cancer stage 4 were found to have a significant positive association (p < 0.05). However, after false discovery rate (FDR) correction none of these associations remained significant at a = 0.05 (Table 1).

### Tissue metabolites & CRG

To explain the variation in CRG (S1 Fig) we combined the clinical covariates with the metabolite abundances and defined them as independent variables and CRG as a response variable, and fitted them an orthogonal partial least squares (OPLS) multivariable regression model (model diagnostics in S2 Fig).

### Benign tissue metabolites & CRG

**Fig 2:**
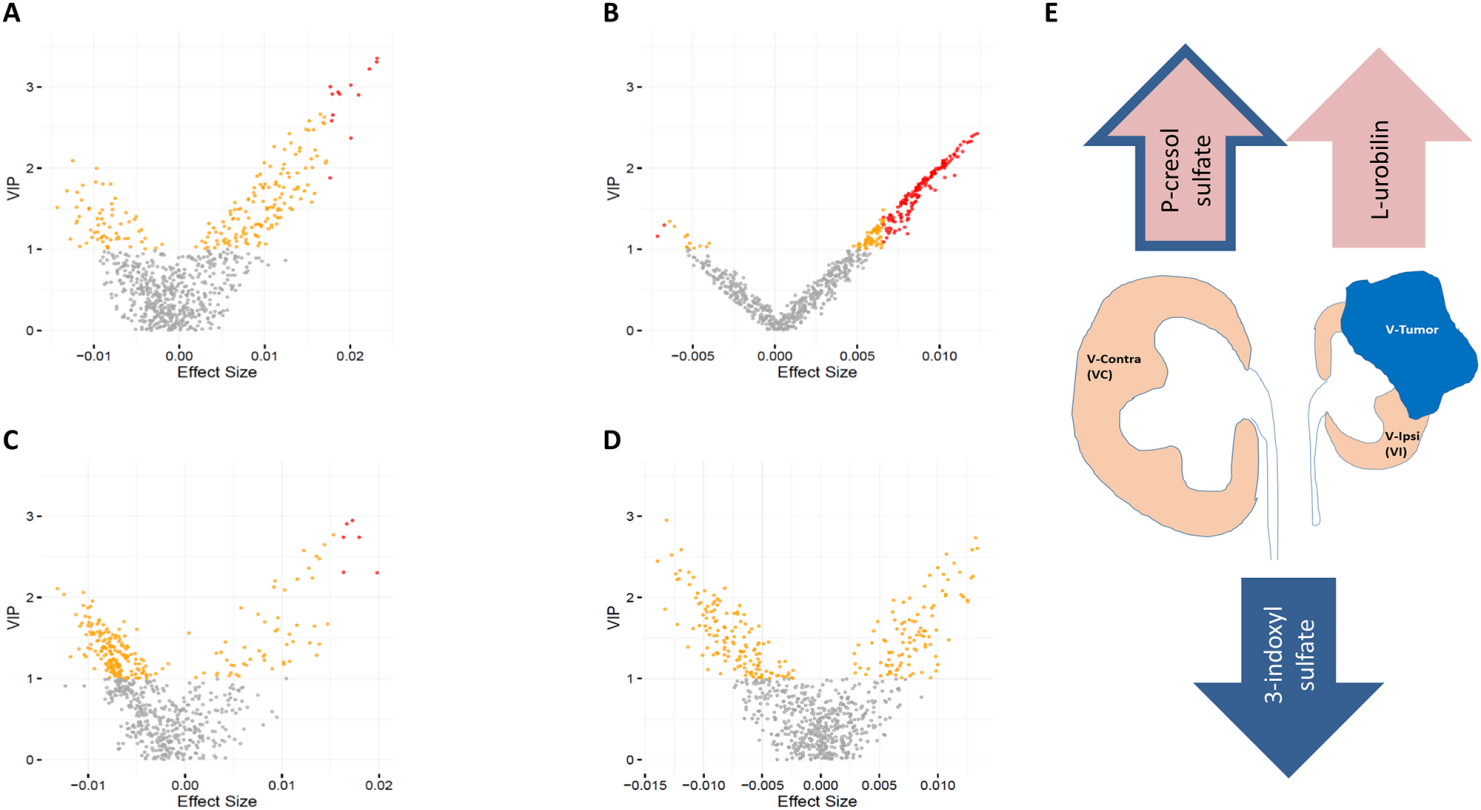
Association of metabolite abundances with compensatory renal growth (CRG). The X-axis is the effect size of the association of metabolite abundance with CRG obtained from the OPLS analysis and the Y-axis is the OPLS VIP score. Panels A and B describe OPLS fit to metabolite abundances in benign and tumor tissue, respectively, with pre-operative CRG as response. Panel C describes OPLS fit to benign/tumor metabolite abundance fold change with pre-operative CRG as response. Panel D describes OPLS fit to metabolite abundances in benign tissue with LT-CRG as response. Statistically significant variables are defined as those with VIP score > 1 and FDR adjusted p-value (p’-value) < 0.05 and are colored red. Variables with VIP score > 1 and p’-value > 0.05 are colored orange, and the remaining variables are colored gray. Panel E describes the associations of a sample of metabolites with pre-operative CRG. Arrows facing up represent a positive association and arrows facing down represent a negative association. The pink arrow represents metabolites within the benign tissue, the blue arrow represents metabolites within the tumor tissue, and the pink arrow with the blue outline represents the benign/tumor metabolite fold-change.

In the benign tissue, 13 metabolites were found to have a significant positive association with CRG (Fig 2A), eight of them identified (S2 Table). Among these is L-urobilin (VIP score = 3.02, adjusted p = 0.017), produced by reabsorption and subsequent oxidation of bilirubin. Interestingly, bilirubin is a recently identified potential risk factor for renal function and CKD progression, where higher bilirubin levels were reported as predictors of favorable renal function outcome [14]. In the tumor tissue, 163 variables were found to significantly associate with CRG, 121 of them are identified metabolites (S2 Table). Only one of them, 3-indoxyl-sulfate (VIP score = 1.3, adjusted p = 0.044), was found to have a negative association with CRG (Fig 2B). 3-indoxyl-sulfate was previously identified as a uremic toxin whose kidney uptake is facilitated by organic anion transporters [15] and is suggested to play a systemic role in CKD [16]. It was additionally shown to suppress proliferation of endothelial progenitor cells (EPCs) [17], indicating a possible connection between derepression of tumor angiogenesis and CRG.

**Fig 3:**
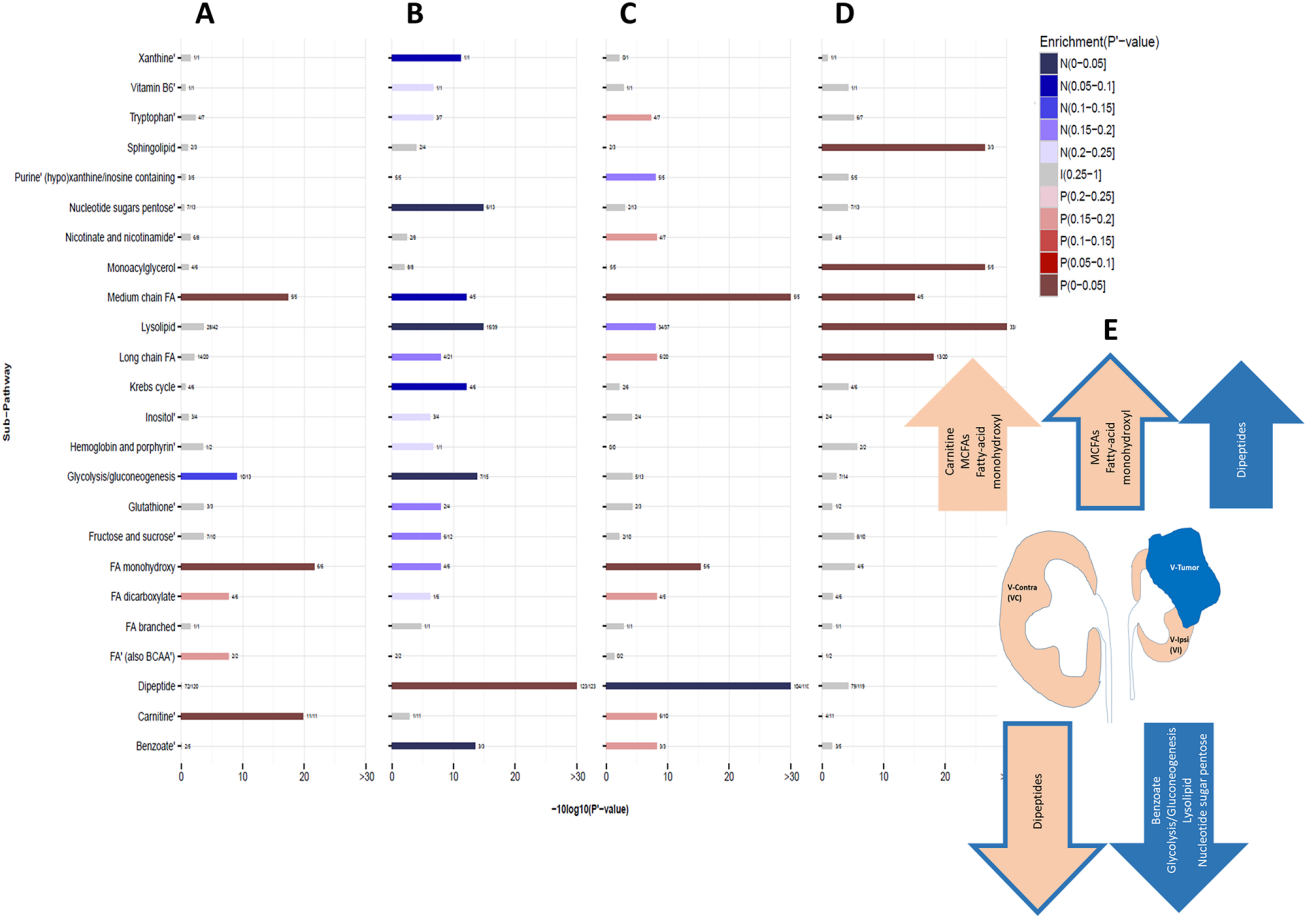
Metabolic sub-pathways enriched in pre-operative compensatory renal growth (CRG). The X-axis is the FDR adjusted p-value (p’-value) of the sub-pathway enrichment analysis, -10log10 transformed, and the Y-axis is the sub-pathway which the metabolites correspond to. In red shades are sub-pathways enriched in metabolites whose abundances are positively associated with CRG (“P” in the legend indicating positive), and in blue shades are sub-pathways enriched in metabolites whose abundances are negatively associated with CRG (“N” in the legend indicating negative). All other sub-pathways (indicated with “I” for invariant in the legend) are colored in gray. Panels A and B describe OPLS fit to metabolite abundances in benign and tumor tissue, respectively, with pre-operative CRG as response. Panel C describes OPLS fit to benign/tumor metabolite abundance fold-change with pre-operative CRG as response. Panel D describes OPLS fit to metabolite abundances in benign tissue with LT-CRG as response. Panel E describes a sample of sub-pathways enriched with metabolites strongly associated with pre-operative CRG. Arrows facing up represent sub-pathways enriched with metabolites with a positive association and arrows facing down represent sub-pathways enriched with metabolites with a negative association. The pink arrow represents sub-pathways enriched with metabolites within benign tissue, the blue arrows represent sub-pathways enriched with metabolites within the tumor tissue, and the pink arrows with the blue outline represent sub-pathways enriched with metabolites from the benign/tumor metabolite fold-change.

We next tested whether specific biological sub-pathways are significantly enriched with metabolites strongly associated with CRG. In the benign tissue, this revealed enrichment of positive associations of carnitine metabolism, medium-chain fatty acids (MCFAs), and fatty-acid monohydroxyl sub-pathways (FDR adjusted p < 0.05) (Fig 3A and 3E). Carnitine is a cofactor required for transport of long-chain fatty acids (LCFAs) into the mitochondrial matrix where they undergo [beta]-oxidation for cellular energy production [18]. MCFAs have been shown to enter the mitochondria independently of carnitine transport but also undergo preferential oxidation for cellular energy production [19]. In the skin, monohydroxy fatty acids were reported to incorporate into the membrane inositol phospholipids [20], historically suggested to play an active role in CRG [21].

### Malignant tissue metabolites & CRG

In the tumor tissue, significant enrichment of dipeptides (positive association) and benzoate, glycolysis/gluconeogenesis, lysolipid and nucleotide sugar pentose (negative associations) sub-pathways were observed (FDR adjusted p < 0.05) (Fig 3B and 3E). Dipeptides may be produced through protein degradation/reutilization processes, and were reported to be elevated in renal tumor tissue at an advanced cancer stage [8].

### Benign/malignant tissue metabolites fold change & CRG

Notwithstanding, these analyses may miss metabolites whose benign versus tumor anti-correlated fold changes are significantly associated with CRG. We thus fitted the OPLS model to the benign/tumor fold change in metabolite abundances, in addition to the relevant clinical covariates (Table 1), as independent variables (model diagnostics in S2C and S2G Fig). This revealed three additional metabolites with a significant positive association with CRG (S2C Fig). Among them is p-cresol sulfate (VIP score = 2.945, adjusted p = 0.033), which was shown to suppress proliferation of EPCs and their angiogenesis capacity [17]. An additional analysis (Methods) shows that the association of p-cresol sulfate with CRG is more strongly contributed by its negative association from the tumor tissue than its positive association from the benign tissue, supporting a connection between derepression of tumor angiogenesis and CRG. Sub-pathway enrichment analysis of these data supported the results of the benign and tumor individual analyses, with positive association of MCFAs and fatty-acid monohydroxy and negative association of dipeptides (FDR adjusted p < 0.05) (Fig 3C and 3E). Albeit, these results should be interpreted cautiously as the OPLS model weakly fitted these data.

### Benign tissue metabolites & long term CRG

Finally, we tested the effect of metabolite abundances in the benign tissue on LT-CRG (model diagnostics in S2D Fig). As LT-CRG was measured only for 22 patients none of the metabolites or covariates met our criteria of statistical significance (Fig 2D). Nevertheless, the sub-pathway enrichment analysis provided further support for positive association of fatty-acid metabolism with CRG, where sphingolipid, monoacylglycerol, LCFAs, and MCFAs are enriched for a negative association with CRG (FDR adjusted p < 0.05) (Fig 3D).

## Discussion

Recent reports correlated clinical covariates, such as age and tumor size with pre-operative CRG [5]. In our analysis no clinical covariate remained significant after FDR correction, emphasizing the need to look beyond conventional clinical and pathologic features such as metabolic processes.

A major hypothesis underlying physiological changes in renal tissue undergoing CRG suggests that the increased need for renal function requires increased production of mitochondrial ATP [22]. Renal proximal tubular cells have been shown to undergo a series of structural changes under CRG [23]. These high energy demanding cells produce ATP in the mitochondria by oxidation of fatty acids and other molecules [18], and indeed it was suggested that following compensatory renal hypertrophy mitochondria show significant acceleration of respiratory activity indicative of a hypermetabolic state [24]. Interestingly, our pre-operative CRG analysis showed enrichment of sub-pathways positively associated with fatty-acid metabolism, fatty-acid monohydroxyl, and both the carnitine dependent and the MCFA sub-pathways, serving to produce acetyl-CoA for Krebs cycle [18,19]. Furthermore, our data suggest that akin to how benign renal histology at nephrectomy predicts LT kidney function [25], the metabolic state of the benign tissue at that time point predicts LT-CRG. This includes MCFAs (also enriched in pre-operative CRG), sphingolipids (related to cell membrane metabolism in CRG [21]), lysolipids (phospholipid breakdown, related to membrane remodeling [26]), and LCFAs and monoacylglycerols (both products of triacylglycerol hydrolysis). Strikingly, all benign tissue metabolites with a significant positive association with pre-operative CRG showed a negative association with LT-CRG, albeit a non-significant one (Fig 2A and 2D, respectively). A possible explanation for this may be that CRG potential is exhausted prior to surgery. Generally, the enrichment of benign tissue metabolic sub-pathways associated with fatty-acid metabolism is in line with recent reports describing that defects in fatty-acid oxidation play a major role in the pathogenesis of chronic kidney disease [18]. Finally, that L-urobilin is significantly positively associated with CRG expands the link between higher levels of heme derivatives and improved kidney function [14]. Boon et al. suggested that endogenously elevated bilirubin specifically protects the vascular compartment from systemic oxidative stress, which potentially clarifies its role in kidney function protection [27]. The association we found between L-urobilin and CRG suggests an additional mechanism for the favorable effect of bilirubin.

In the tumor tissue a far higher number of metabolites were found to significantly associate with CRG. Although this may be partially explained by higher variability in tumor metabolite abundances providing more statistical power, it also shifts the focus from benign mass reduction alone.

The enrichment of the dipeptides (positive association) and benzoate, glycolysis/gluconeogenesis, lysolipid and nucleotide sugar pentose (negative association) sub-pathways among tumor metabolites strongly associated with CRG (Fig 3B and 3E) supports a previous report demonstrating dipeptides and glycolysis pathways to characterize tumor tissue [8]. The novelty here is that the magnitudes of these processes are significantly associated with CRG (positive for dipeptides and negative for others). Their contrasting associations suggest that the tumor-CRG interaction is more complex than progression dependent alone.

Interestingly, we recently reported higher levels of dipeptides to correlate with a higher cancer stage [8]. These findings are in line with our current findings, correlating to some extent advanced cancer stage with CRG (p < 0.05). Furthermore, several specific metabolites, such as galactose (VIP score = 1.78, adjusted p = 0.005) and asparagine (VIP score = 1.274, adjusted p = 0.016) which are also associated with CRG, were previously reported by our group to correlate with advanced cancer stage. Huaser et al. reported that GALE suppression (UDP-glucose 4-epimerase, catalyzing the final step in the Leloir pathway of galactose metabolism), occurs in uni-nephrectomized rats. They suggested that the suppression of such regulating genes, responsible predominantly for growth inhibition and apoptosis, results in a net pro-hypertrophic response [28].

Our current analysis found a negative association between the glycolysis/gluconeogenesis pathway in the tumor tissue and CRG. We recently reported an increase in abundance of metabolites of the upper glycolysis pathway alongside a decrease in the abundance of metabolites in the lower glycolysis pathway in clear cell RCC, suggesting a shift towards the pentose phosphate pathway [8]. However, a detailed analysis of metabolite abundance and CRG did not reveal similar trends. An additional distinct tumor pathway analysis showed that elevated glutathione levels correlate with advanced cancer stage(8). Interestingly, previous publications correlated glutathione transporters [24], increased cellular synthesis and content of glutathione [29], with CRG. However, our current analysis did not find an association of the glutathione pathway with CRG. Altogether these findings may suggest several elements of advanced disease metabolism, where not all are associated with CRG.

That the Lysolipid pathway in the tumor tissue is negatively associated with CRG, whereas the opposite direction of association was found in the benign tissue, may further support a crosstalk between the benign and malignant tissue with respect to CRG, which is intriguing considering the importance of membrane remodeling in CRG [26].

Finally, of all tumor metabolites significantly associated with CRG, only 3-indoxyl-sulfate was found to have a negative effect. This metabolite and p-cresol sulfate were both suggested to repress angiogenesis [17,30], hence our finding that the two are negatively associated with CRG strongly indicates that the benign kidney tissue responds to the tumor’s metabolic state. That both of these uremic toxins have a systemic effect echoes the idea of a “renotrophic” “internalist’s tumor”.

Notwithstanding our results, our study has several limitations. First, our cohort is heterogeneous including both partial and radical nephrectomy patients. However, as most of the analysis included pre-operative CRG, type of surgery that followed should not entail major bias. Second, the fact that our tissue samples were collected from only the tumor bearing kidney may mean that our results do not faithfully represent the bilateral CRG process. This limitation, however, is practically inevitable due to the impracticality of acquiring tissue from a contralateral kidney for prospective studies. Also, a similar approach is often applied in diagnostic biopsies for kidney disease. Last, our study is considerably underpowered in terms of sample size versus the number of variables, which stems from the fact that obtaining human samples is a big challenge. However, the fact that the OPLS model, which is designed to handle such designs, reported statistically significant results indicates a trend in the data and hence addition samples should not only amplify it but may also reveal additional metabolites and biological pathways that are significantly associated with CRG.

## Conclusion

Our work identifies benign parenchymal- and tumor-specific metabolites and metabolic sub-pathways strongly associated with CRG, which therefore may be driving it. This deepens our understanding of kidney pathophysiology [18] and provides a new perspective on CRG.

## Supporting information

S1 Fig Distribution of compensatory renal growth (CRG). Distribution of CRG across the cohort of 49 patients.
(TIF)

S2 Fig OPLS model fit diagnostics. QQ-plots of the OPLS fits and root mean squared errors (RMSE) computed for the 8-fold cross-validation OPLS fits and an OPLS fitted to the randomized data. Panels A and B and E and F describe OPLS fit to metabolite abundances in benign and tumor tissue, respectively, with pre-operative CRG as response. Panels C and G describe OPLS fit to benign/tumor metabolite abundance fold change with pre-operative CRG as response. Panels D and H describe OPLS fit to metabolite abundances in benign tissue with LT-CRG as response. P-values in panels E-H are the statistical significance of comparing each pair of corresponding cross-validation and randomized RMSEs. In addition, the R^2^ and Q^2^ values of each model fit are also reported in panels E-H.
(TIF)

S3 Fig Distribution of observer1/observer2 fold change of compensatory renal growth (CRG) measurements. Compensatory renal growth (CRG) was measured for 42 patients by two observers and the histogram of the fold change of their CRG measurements identifies two patients for which the CRG measurement of observer 1 was more than 25% than that of observer 1 (to the right of the right dashed vertical line) and one patient for which the CRG measurement of observer 1 was less than 75% than that of observer 1 (to the left of the left dashed vertical line).
(TIF)

S4 Fig The statistical significance of the effect of metabolite abundances on compensatory renal growth (CRG). The X-axis is the FDR adjusted p-value (p’-value) based on a univariable regression and the Y-axis is the OPLS VIP score. Panels A and B describe OPLS fit to metabolite abundances in benign and tumor tissue, respectively, with pre-operative CRG as response. Panel C describes OPLS fit of benign/tumor metabolite abundance fold change with pre-operative CRG as response. Panel D describes OPLS fit of metabolite abundances in benign tissue with LT-CRG as response. Statistically significant variables are defined as those with VIP score > 1 and p’-value < 0.05 and are colored red. Variables with VIP score > 1 and p’-value > 0.05 are colored orange, and the remaining variables are colored gray.
(TIF)

S1 Table The Cohort sheet provides the covariate and metabolite abundances data for the cohort of 138 patients. Metabolite abundances in benign, tumor, and benign/tumor fold change are indicated by their compound ID (e.g., 38002_benign, 38002_tumor, and 38002_benign_tumor_fc, respectively). The Metabolites sheet provides the metadata for each metabolite by its compound ID, such as biochemical name and sub-pathway.
(XLSX)

S2 Table S2 Table has four sheets corresponding to the four analyses described in the manuscript: Association of benign metabolite abundances with pre-operation CRG, association of tumor metabolite abundances with pre-operation CRG, association of benign/tumor metabolite abundance fold change with pre-operation CRG, and association of benign metabolite abundances with LT-CRG. In each sheet, the univariable and OPLS analyses are provided for each variable in terms of its effect and the associated statistical significance.
(XLSX)
