# Supplementary figures and images for: Benign and tumor parenchyma metabolomic profiles affect compensatory renal growth in renal cell carcinoma surgical patients
Source: PLoS One. 2017 Jul 20;12(7):e0180350. doi: 10.1371/journal.pone.0180350 (PMC5519040; doi:10.1371/journal.pone.0180350)

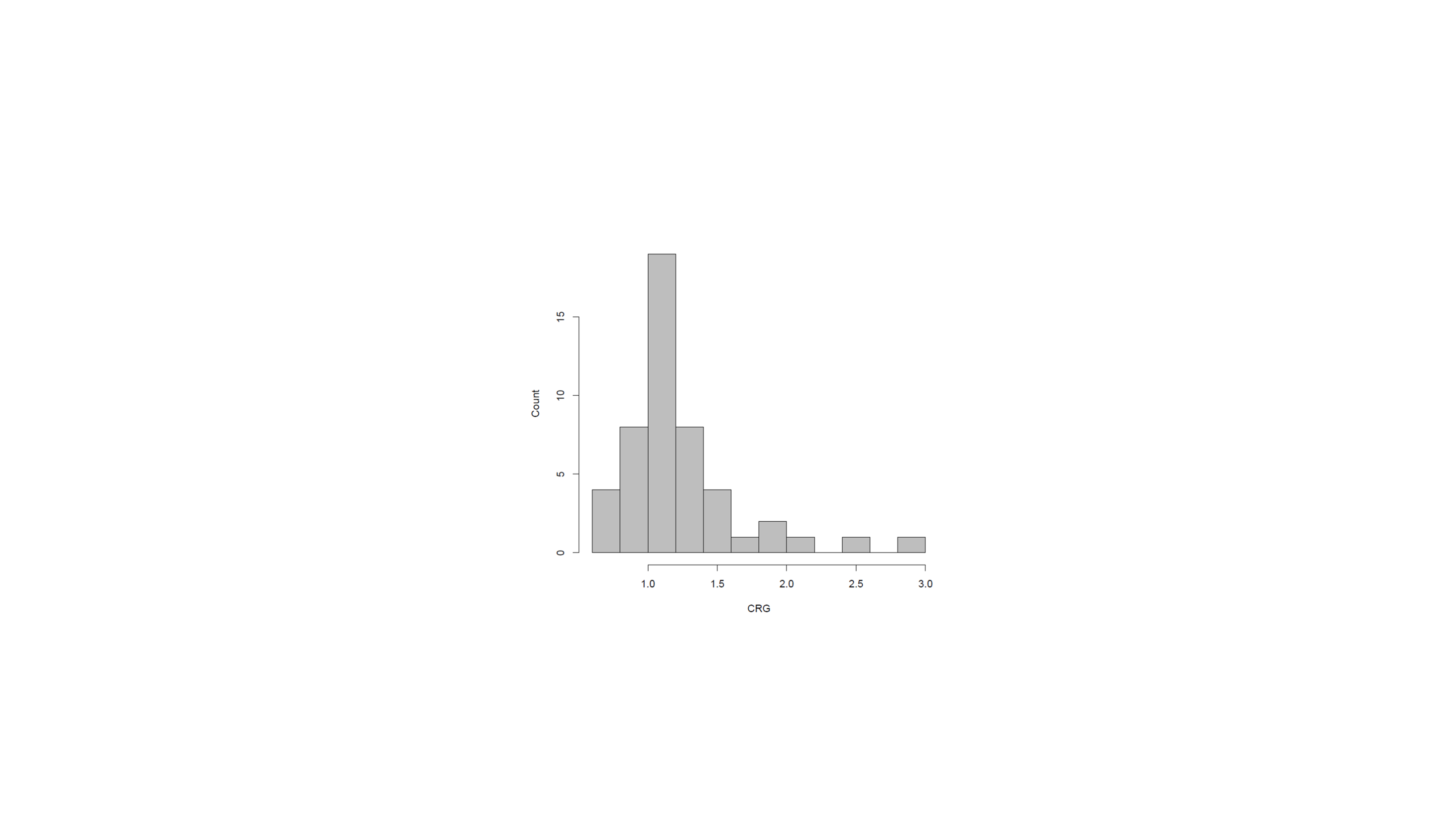

Supplement: S1 Fig — Distribution of CRG across the cohort of 49 patients. (TIF) [file pone.0180350.s001.tif]

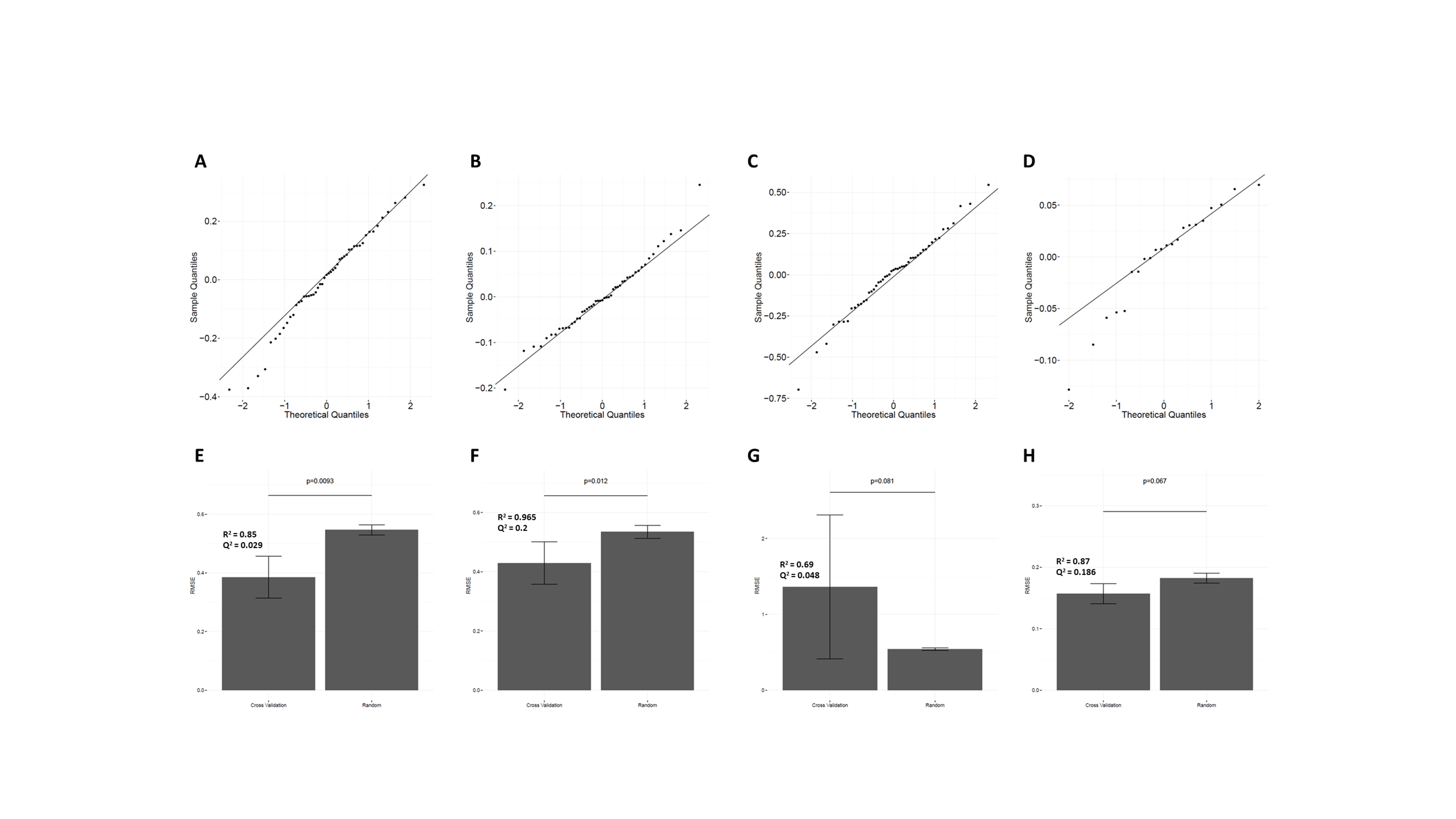

Supplement: S2 Fig — QQ-plots of the OPLS fits and root mean squared errors (RMSE) computed for the 8-fold cross-validation OPLS fits and an OPLS fitted to the randomized data. Panels A and B and E and F describe OPLS fit to metabolite abundances in benign and tumor tissue, respectively, with pre-operative CRG as response. Panels C and G describe OPLS fit to benign/tumor metabolite abundance fold change with pre-operative CRG as response. Panels D and H describe OPLS fit to metabolite abundances in benign tissue with LT-CRG as response. P-values in panels E-H are the statistical significance of comparing each pair of corresponding cross-validation and randomized RMSEs. In addition, the R2 and Q2 values of each model fit are also reported in panels E-H. (TIF) [file pone.0180350.s002.tif]

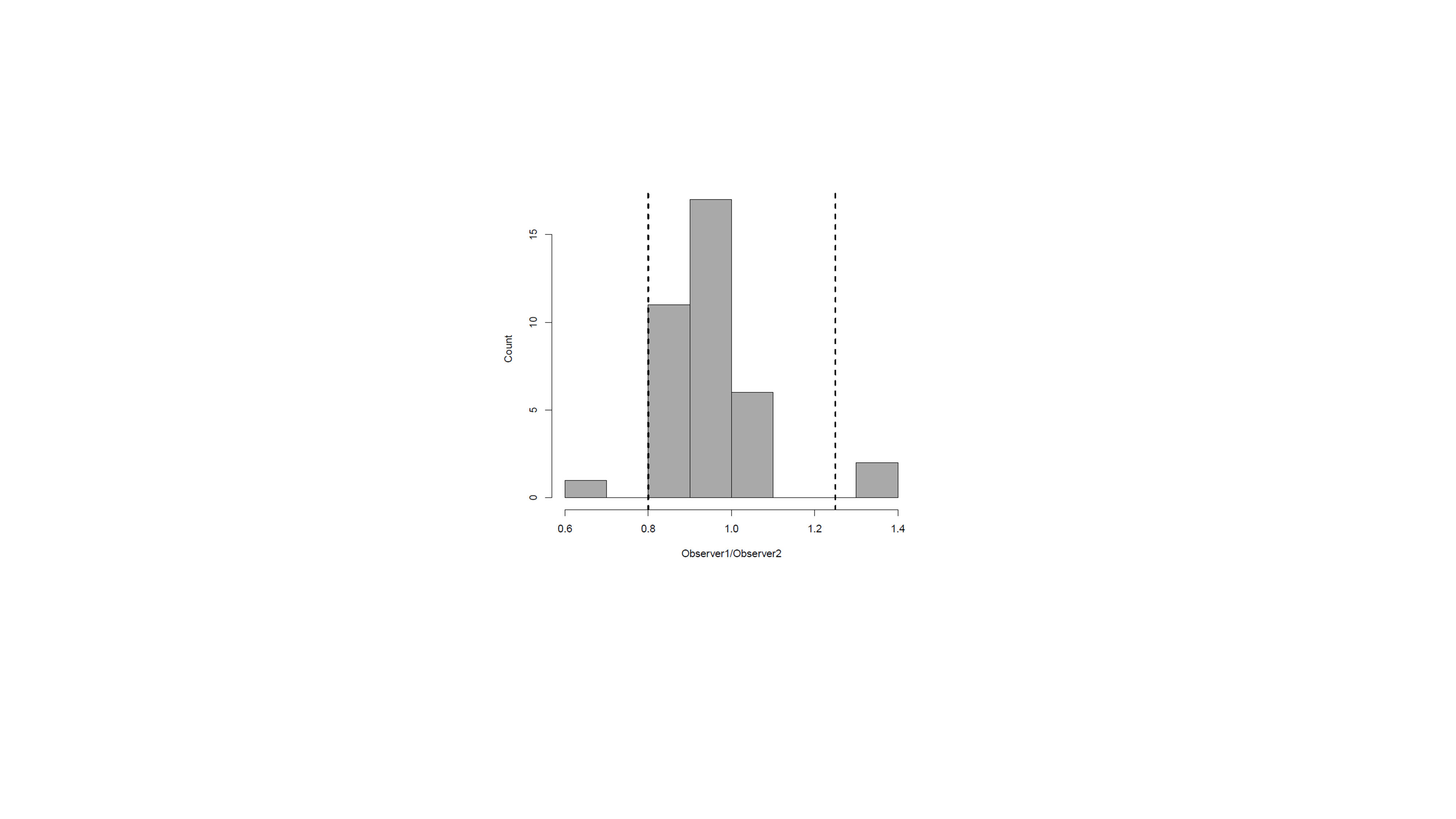

Supplement: S3 Fig — Compensatory renal growth (CRG) was measured for 42 patients by two observers and the histogram of the fold change of their CRG measurements identifies two patients for which the CRG measurement of observer 1 was more than 25% than that of observer 1 (to the right of the right dashed vertical line) and one patient for which the CRG measurement of observer 1 was less than 75% than that of observer 1 (to the left of the left dashed vertical line). (TIF) [file pone.0180350.s003.tif]

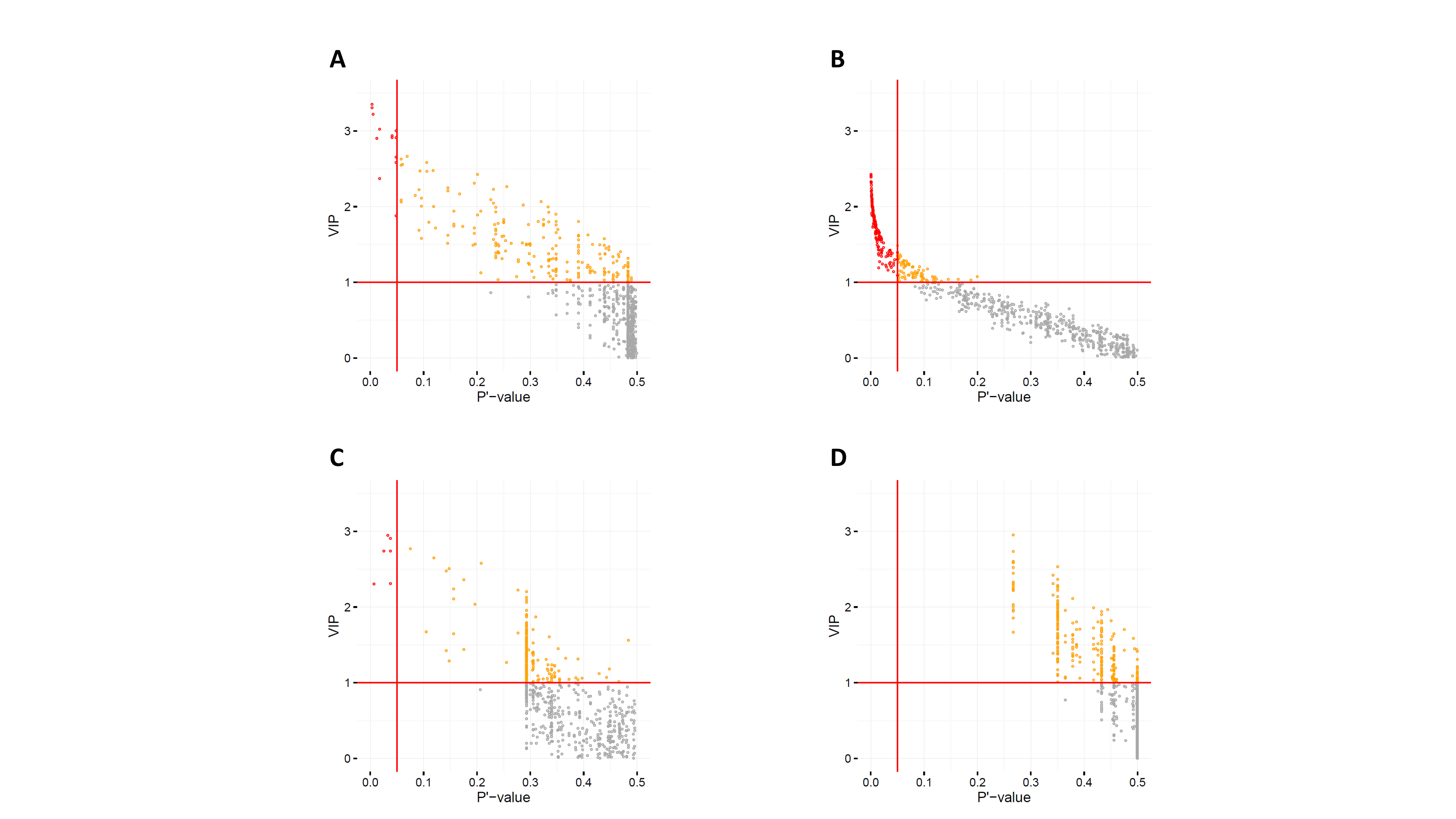

Supplement: S4 Fig — The X-axis is the FDR adjusted p-value (p’-value) based on a univariable regression and the Y-axis is the OPLS VIP score. Panels A and B describe OPLS fit to metabolite abundances in benign and tumor tissue, respectively, with pre-operative CRG as response. Panel C describes OPLS fit of benign/tumor metabolite abundance fold change with pre-operative CRG as response. Panel D describes OPLS fit of metabolite abundances in benign tissue with LT-CRG as response. Statistically significant variables are defined as those with VIP score > 1 and p’-value < 0.05 and are colored red. Variables with VIP score > 1 and p’-value > 0.05 are colored orange, and the remaining variables are colored gray. (TIF) [file pone.0180350.s004.tif]
